# Long-Term Outcome of Temporal Lobe Epilepsy Surgery in 621 Patients With Hippocampal Sclerosis: Clinical and Surgical Prognostic Factors

**DOI:** 10.3389/fneur.2022.833293

**Published:** 2022-04-25

**Authors:** Marina Teixeira Ramalho Pereira Dalio, Tonicarlo Rodrigues Velasco, Izabela Dayany Franca Feitosa, João Alberto Assirati Junior, Carlos Gilberto Carlotti Junior, João Pereira Leite, Antonio Carlos dos Santos, Veriano Alexandre, Frederico Nakane Nakano, Ricardo Lutzky Saute, Lauro Wichert-Ana, Americo Ceiki Sakamoto

**Affiliations:** ^1^Epilepsy Surgery Center (CIREP), Department of Neurosciences and Behavioral Sciences, Hospital of Clinics of the Medical School of Ribeirão Preto of the University of São Paulo (HCFMRP-USP), Ribeirão Preto, Brazil; ^2^Department of Surgery and Neurosurgery, Hospital of Clinics of the Medical School of Ribeirão Preto of the University of São Paulo (HCFMRP-USP), Ribeirão Preto, Brazil; ^3^Department of Radiology, Hospital of Clinics of the Medical School of Ribeirão Preto of the University of São Paulo (HCFMRP-USP), Ribeirão Preto, Brazil

**Keywords:** temporal lobe epilepsy, hippocampal sclerosis, epilepsy surgery, surgical outcome, surgical prognostic factors, long-term outcome

## Abstract

Temporal lobe epilepsy (TLE) is the most common type of focal epilepsy and is frequently drug-resistant (DR) to antiseizure medication (ASM), corresponding to approximately one-third of the cases. When left inadequately treated, it can worsen the quality of life, cognitive deficits, and risk of death. The standard treatment for drug-resistant TLE is the surgical removal of the structures involved, with good long-term outcome rates of 60–70 % and a low rate of adverse effects. The goal of successful treatment is sustained seizure freedom. In our study, we evaluated sustained long-term (up to 23 years) surgical outcomes in 621 patients with DR-TLE associated with hippocampal sclerosis, who underwent a temporal lobectomy. We analyzed the main predictive factors that influence the surgical outcome related to seizure control, through a longitudinal and retrospective study, using a multivariable regression model. We found that 73.6% of the patients were free from disabling seizures (Engel Class I), maintained over time in 65% of patients followed up to 23 years after surgery. We found that four independent variables predicted seizure outcomes. The presence of dysmnesic and olfactory aura predicted a less favorable outcome. The history of febrile seizure and the surgical technique predicted a good outcome. Regarding the type of surgical technique, the standard anteromesial temporal lobectomy (ATL) led to significantly better outcomes (78.6% Engel Class I) when compared to the selective amygdalohippocampectomy *via* subtemporal approach (67.2% Engel Class I; *p* = 0.002), suggesting that the neuronal networks involved in the epileptogenic zone may be beyond mesial temporal structures. The multivariable regression model with the above-mentioned predictor variables revealed an ExpB = 3.627 (*N* = 621, *p* < 0.001), indicating that the model was able to distinguish between patients with a seizure-free. We conclude that epilepsy surgery is a safe procedure, with low rates of postoperative complications and good long-term results.

## Introduction

Epilepsy is a chronic neurological disease that affects 1% of the world's population (1). It is considered a public health problem, responsible for ≈0.5% of the costs of disease burden (2). Temporal lobe epilepsy (TLE) is one of the commonest types, corresponding up to 35% of all epilepsies (3), and is frequently drug-resistant (DR) to antiseizure medication (ASM) (4). When left uncontrolled, drug-resistant epilepsy (DRE) can worsen the quality of life, cognitive deficits, and increase the risk of death 5–10 times higher than the general population (5). It is well known that epilepsy surgery is a safe and efficient treatment for DR-TLE (6–10), with a low rate of adverse effects (11). The benefits of the surgery are a decrease in frequency and severity of seizures, a decrease in mortality, a better quality of life, and higher rates of return to school and work (8, 10).

Long-term seizure outcome studies have reported success rates of 60–70% (12, 13). However, fewer studies were conducted with enough sample size (12). In a recent Cochrane review, published in 2019, the evidence regarding seizure outcome was considered moderate to low mostly due to small sample sizes (11). The authors suggested that future research should be appropriately powered, focus on issues related to the site-specific surgical approach, extent of resection, and should investigate the prognostic factors related to the outcome of surgery *via* multivariable statistical regression modeling.

Here, we evaluated the sustained long-term surgical outcomes (up to 23 years) in 621 patients with DR-TLE associated with hippocampal sclerosis. We analyzed the most important predictive factors of surgical success related to seizure control using a multivariable regression model. We also reported adverse events after surgery. We found that after more than 20 years of follow-up, 65% of patients were seizure-free after surgery. We also found that resection of the temporal pole along with amygdalohippocampectomy significantly improved the chances of being seizure-free in the long term, when compared to selective amygdalohippocampectomy.

## Methods

Longitudinal and retrospective data were collected in a homogeneous sample of patients with drug-resistant temporal lobe epilepsy (DR-TLE) associated with hippocampal sclerosis (HS), who underwent a temporal resection at our Epilepsy Surgery Center (CIREP) in the Hospital of Clinics of the Medical School of Ribeirão Preto of the University of São Paulo (HCFMRP-USP), Brazil, between 1994 and 2011. We evaluated postsurgical clinical follow-up data up to a maximum of 23 years (mean: 11.6 years ± 5.3). We selected patients over 18 years at the time of surgery with a clinical diagnosis of temporal lobe epilepsy and magnetic resonance imaging (MRI) suggestive of hippocampal sclerosis, namely hippocampal atrophy, loss of internal structure, and decreased T1 and increased T2 signal intensity. MRI images were acquired using a Magneton Vision 1.5 T scanner (Siemens) or a Phillips Activa 3 T scanner (after 2007), with special protocols for epilepsy evaluation. The exclusion criteria were: (i) the presence of a lesion different from hippocampal sclerosis on neuropathological examination, such as cortical dysplasia or malformations of cortical development; (ii) the extra-temporal or multilobar resection; (iii) MRI-negative patients; and (iv) those who lost follow-up before 1 year, or lack of information in the medical record. The group of senior neurophysiologists was the same throughout the study.

The information was obtained by reviewing medical records and through a telephone interview. We collected the following data: clinical history, preoperative evaluation, and postoperative clinical follow-up.

Phase I of the presurgical evaluation in our epilepsy surgery center has been previously published elsewhere (14). It consisted of MRI, Video Electroencephalogram (VEEG), social, neuropsychological, and psychiatric evaluations. The probable epileptogenic zone was defined in a multidisciplinary meeting. When indicated, we performed additional tests in phase II of the presurgical assessment: SPECT (single-photon emission computed tomography), Positron emission tomography (PET), functional MRI, volumetric MRI studies, Intra-carotid amobarbital procedure (WADA) test, semi-invasive evaluation with foramen ovale electrodes, or stereoelectroencephalography (SEEG).

**Figure 1:**
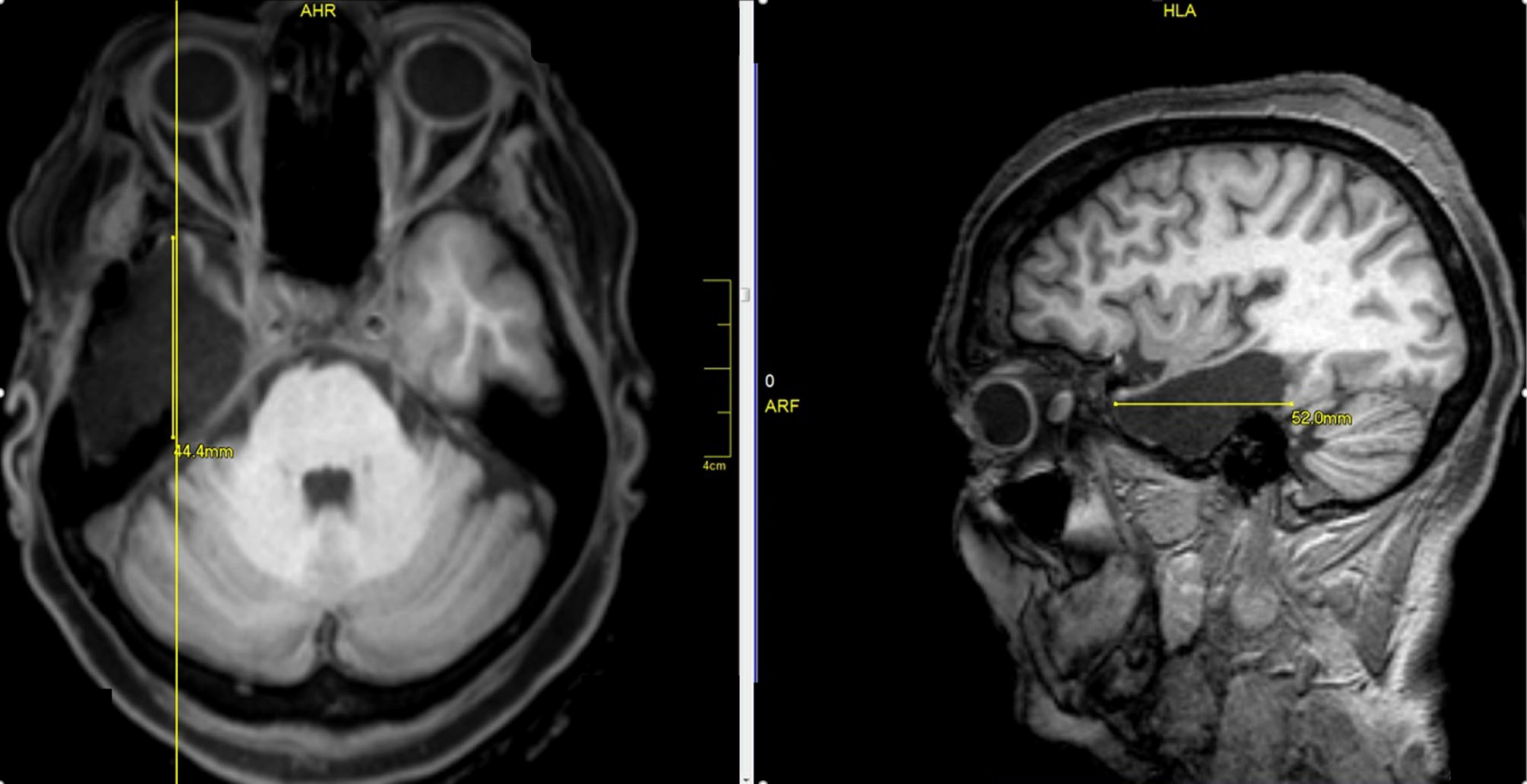
Right standard anteromesial temporal lobectomy with amygdalohippocampectomy.

**Figure 2:**
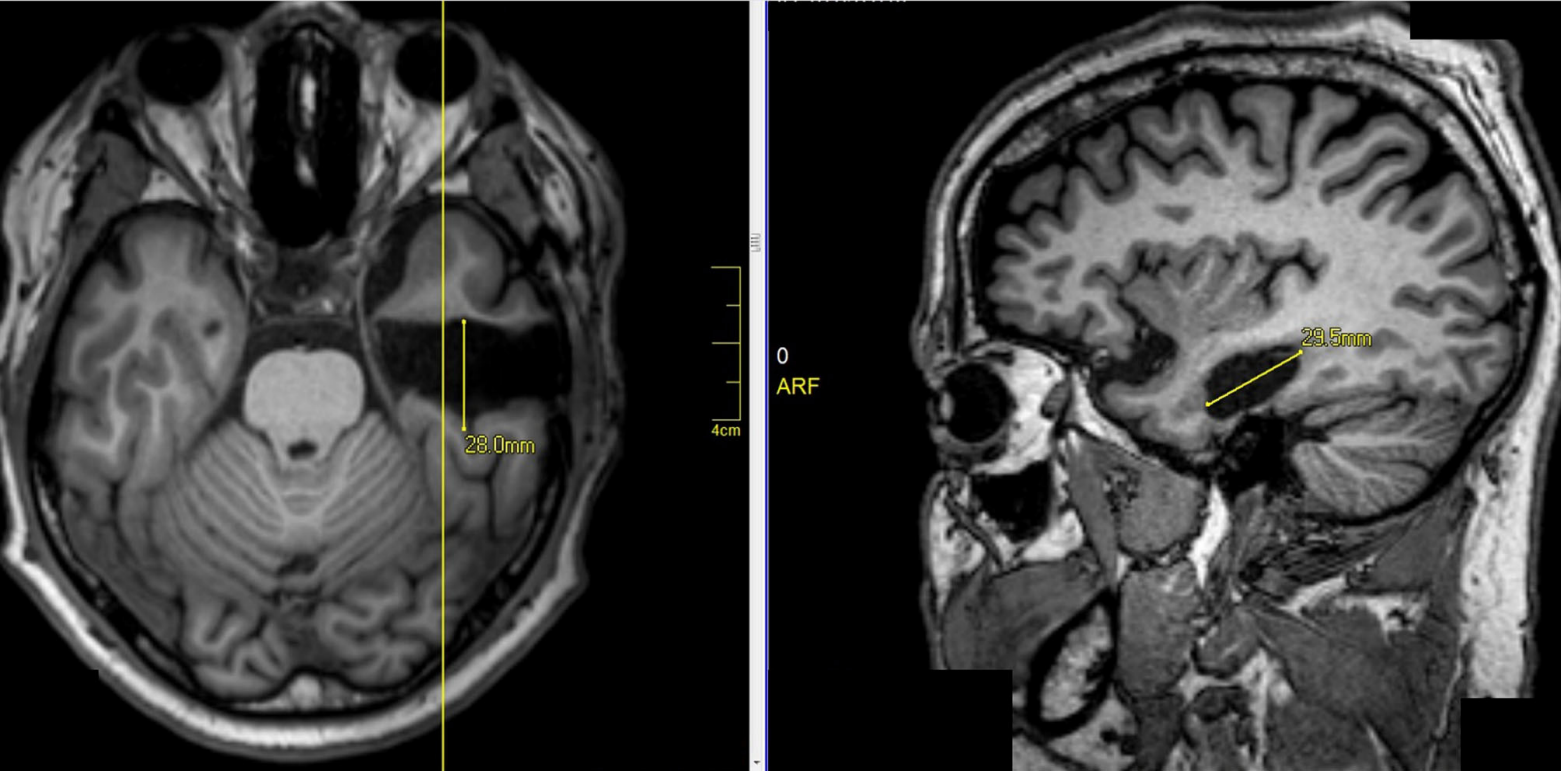
Selective amygdalohippocampectomy (SAH) *via* subtemporal approach, with preservation of the pole.

Two surgeons operated on all patients. Surgeon 1 performed a standard anteromesial temporal lobectomy (ATL) with amygdalohippocampectomy (Figure 1). Surgeon 2 performed a selective amygdalohippocampectomy (SAH) *via* subtemporal approach, with preservation of the temporal pole (Figure 2). Patients were randomly selected to surgeon 1 or surgeon 2, without previous knowledge of presurgical evaluation data.

The surgical outcomes were obtained by regular follow-up visits or telephone interviews. We evaluated seizure outcomes using the Engel scale (7). We also investigated the early and late surgical complications. If the patient was seizure-free after 3 years of follow-up, we offered the possibility of reducing or completely removing the ASM. During the follow-up period, the number of ASM could be reduced in the presence of not-tolerated or serious adverse effects.

The univariate statistical analysis was performed using the following independent variables, such as sex, age of onset of epileptic seizures, epilepsy duration until surgical intervention, follow-up time duration, age at surgery, risk factors for epilepsy, ictal semiology, and lateralization signs, presence of aura, seizure frequency before surgery, number of anti-epileptic drugs used immediately before surgery, number of drug resistances, preoperative neurophysiology, preoperative MRI, brain CT, invasive evaluation, type of surgery: standard anterior temporal lobectomy (with resection of the temporal pole) or selective lobectomy (which preserves the temporal pole), lobectomy side (left or right), presence or absence of complications in the immediate or late postoperative period, time of seizure recurrence in the postoperative period (in months), Engel classification in the last follow-up visit, number of anti-epileptic drugs in the last visit, and whether the patient was reoperated or not. For the dependent categorical variables, we performed “Qui square tests” and “*T*-Test” for the continuous variable. For the dependent variable “Time,” we used the Kaplan–Meier curve to verify the relapse time.

Binary logistic regression was performed to assess the impact of this set of independent (predictor) variables on the odds that patients would become seizure-free after surgery (the dependent variable). We included in the model the independent variables with a *p* < 0.2 in the univariate analysis and the final model *p*-value under 0.05. Our goal was to assess the variables that could predict surgical outcomes and create a model that we can apply to new similar cases in the future, thus being able to explain to the patient more clearly and objectively the probability of being seizure-free after the surgery.

This study was approved by the Research Ethics Committee of HCFMRP-USP.

## Results

We analyzed 637 adult patients with DR-TLE associated with HS, operated between 1994 and 2011. Ten patients were excluded due to a loss of follow-up, five patients were excluded due to non-epileptic seizures, and one patient due to lack of information in the medical record. The sample size at final follow-up was 621 patients, of which 336 were female (54.1%; 95% *CI*: 50.1–58.1%) and 285 were male (45.9%; 95% CI: 41.9–49.9%). Age at onset of epilepsy ranged from 0 to 47 years (mean 12.0 ± 8.5), epilepsy time (duration of epilepsy) ranged from 0.7 to 57.3 years (mean 26.7 years ± 10.85). The age at surgery ranged from 18.6 to 70 years (mean: 38.6 ± 9.4). The risk factors for epilepsy and medical history data are summarized in Table 1.

**Table 1:** Risk factors for epilepsy and medical history.

**Risk factors for epilepsy and medical history**	***N***	**Lack of information**	**Total**	**Percentage**	**Valid percentage**	**CI 95%**
Neurocysticercosis	67	2	619	10.8	10.8	8.5–13.5
Prolonged non-febrile seizures	140	2	619	22.5	22.6	19.4–26.1
Febrile seizures	189	2	619	30.4	30.5	26.9–34.3
Status epilepticus	47	2	619	7.6	7.6	5.6–10.0
Status epilepticus in the evolution	121	2	619	19.5	19.5	16.5–22.9
Meningitis and encephalitis	41	2	619	6.6	6.6	4.8–8.9
Traumatic brain injury (TBI)	44	3	618	7.1	7.1	5.2–9.4
Obstetrict complication	107	8	613	17.2	17.5	14.5–20.7
Delay in neuropsychomotor development	29	7	614	4.7	4.7	3.2–6.7
Presence of previous focal neurological deficit	15	0	621	2.4	2.4	1.4–4.0
Family history of febrile seizures	32	0	621	5.2	5.2	3.6–7.2
Family history of epilepsy	305	0	621	49.1	49.1	45.1–53.1
History of bilateral tonic-clonic seizures	8	0	621	1.3	1.3	0.6–2.5

During the pre-surgical evaluation, 390 patients reported a weekly seizure frequency of (64.8%; 95% *CI*: 60.8–68.6). Regarding seizure semiology, 536 reported the presence of aura (86.3%; 95% *CI*: 83.4–88.9), especially the temporal mesial auras, corresponding to 394 patients (63.4%; 95% *CI*: 59.5–67.2), and epigastric aura (alone) was reported by 252 patients (41.7%; 95% *CI*: 37.7–45.7). The details of the auras are summarized in Table 2.

**Table 2:** Auras.

**Aura**	**N**	**Missing**	**Total N**	**Percentage**	**Valid percentage**	**IC 95%**
Presence of aura	536	0	621	86.3	86.3	83.4–88.9
Denied aura	63	16	605	10.1	10.4	8.1–13.1
Inespecific	69	16	605	11.1	11.4	9.0–14.2
Epigastric	252	16	605	40.6	41.7	37.7–45.7
Affective	106	16	605	17.1	17.5	14.6–20.8
Dismnestic	17	16	605	2.7	2.8	1.6–4.5
Autonomic	31	16	605	5.0	5.1	3.5–7.2
Sensitive	27	16	605	4.3	4.5	3.0–6.4
Visual	9	16	605	1.4	1.5	0.7–2.8
Gustatory	12	16	605	1.9	2.0	1.0–3.4
Olfactory	8	16	605	1.3	1.3	0.6–2.6
Cefalic	20	16	605	3.2	3.3	2.0–5.1
Vertiginous	22	16	605	3.5	3.6	2.3–5.5
Auditory	3	16	605	0.5	0.5	0.1–1.4
Aphasia	1	16	605	0.2	0.2	0–0.9
Mesial temporal	394	16	605	63.4	65.1	59.5–67.2
Lateral temporal	25	16	605	4.0	4.1	2.6–5.9
Extratemporal	36	16	605	5.8	6.0	4.1–7.9

Four hundred and ten patients had at least two drugs confirmed resistances (66.7%; 95% CI: 62.8-70.4), 205 had more than two drug resistances, and in six patients, drug resistance could not be established. At the time of surgery, 521 patients were taking one or two antiseizure medications (85.0%; 95% *CI*: 81.9–87.7), and 100 patients were taking three or more ASMs.

Regarding the presurgical evaluation, 420 patients had normal baseline activity on the EEG (71.6%; 95% *CI*: 67.7–75.2), 449 had unilateral interictal epileptiform discharge ipsilateral to HS (75.3%; 95% *CI*: 71.7–78. 7), 505 had ictal onset EEG in the temporal region ipsilateral to the HS (86.0%; 95% *CI*: 83.0–88.7), and 66 performed invasive evaluation (10.6%; 95% *CI*: 8.3–13.3).

As for neuroimaging evaluation, 600 patients had unilateral HS on the MRI (96.6%; 95% *CI*: 94.9–97.9), 21 had bilateral HS on MRI (3.4 %; 95% *CI*: 2.1–5.1).

Regarding surgical data, 355 patients performed ATL with temporal pole resection (57.5 %; 95% *CI*: 53.5–61.5) and 262 performed SAH *via* subtemporal approach without temporal pole resection (42.5%; 95% *CI*: 38.5–46.5).

### Postsurgical Seizure Outcome Factors

**Figure 3:**
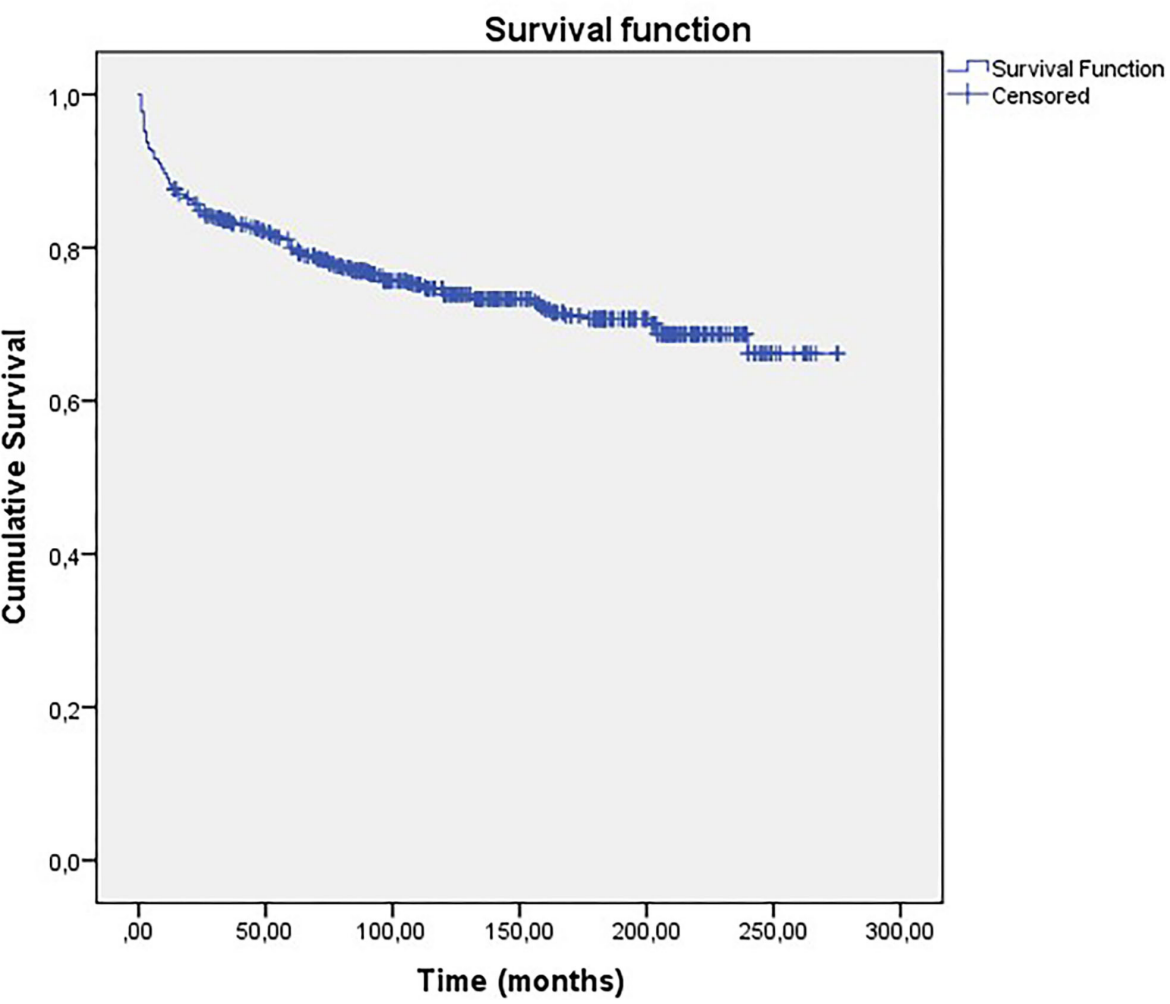
Kaplan-Meier curve of long-term seizure outcomes for all patients.

The mean follow-up duration varied between 1 and 23 years (mean = 11.6 years, ±5.35). The surgical outcome classified by the Engel Scale (7) is summarized in Table 3. Overall, 457 patients were free from disabling seizures at the last follow-up (Engel Class I) (73.6%; 95% *CI*: 69.9–77.0). Figure 3 shows the Kaplan–Meier curve regarding long-term seizure outcomes. Patients followed by more than 20 years had seizure-free rates of ~65%.

**Table 3:** Summarized Engel.

**Frequency**		**Valid percentage**	**CI 95%**
**I**	457	**73.6**	69.9–77.0
II	69	11.1	8.7–13.9
III	86	13.8	11.2–16.8
IV	9	1.4	0.7–2.7
Total	621	100.0	

*bold values is: Engel Class I - free from disabling seizures*.

**Figure 4:**
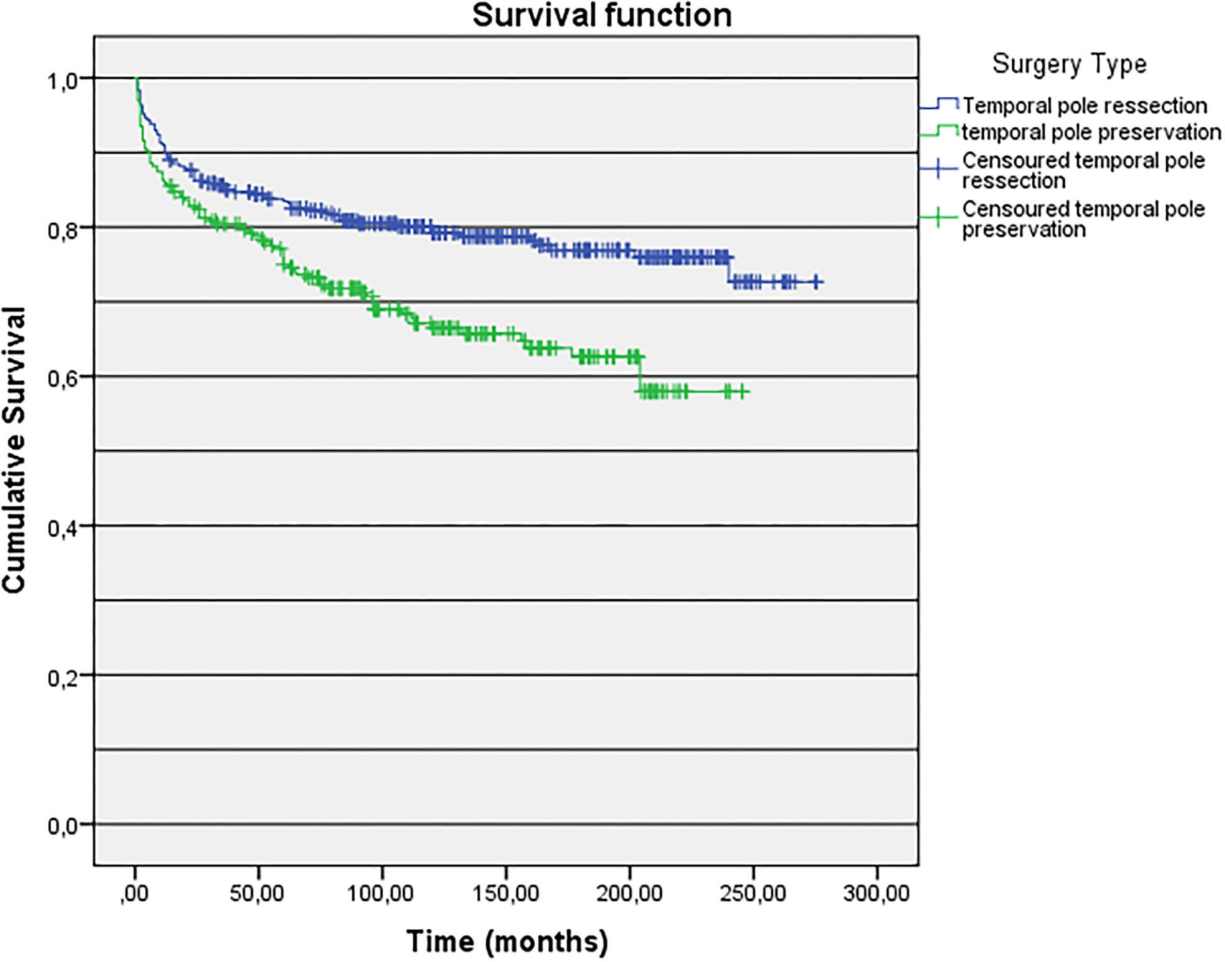
Kaplan-Meier curve of long-term seizure outcomes according to type of surgery.

**Figure 5:**
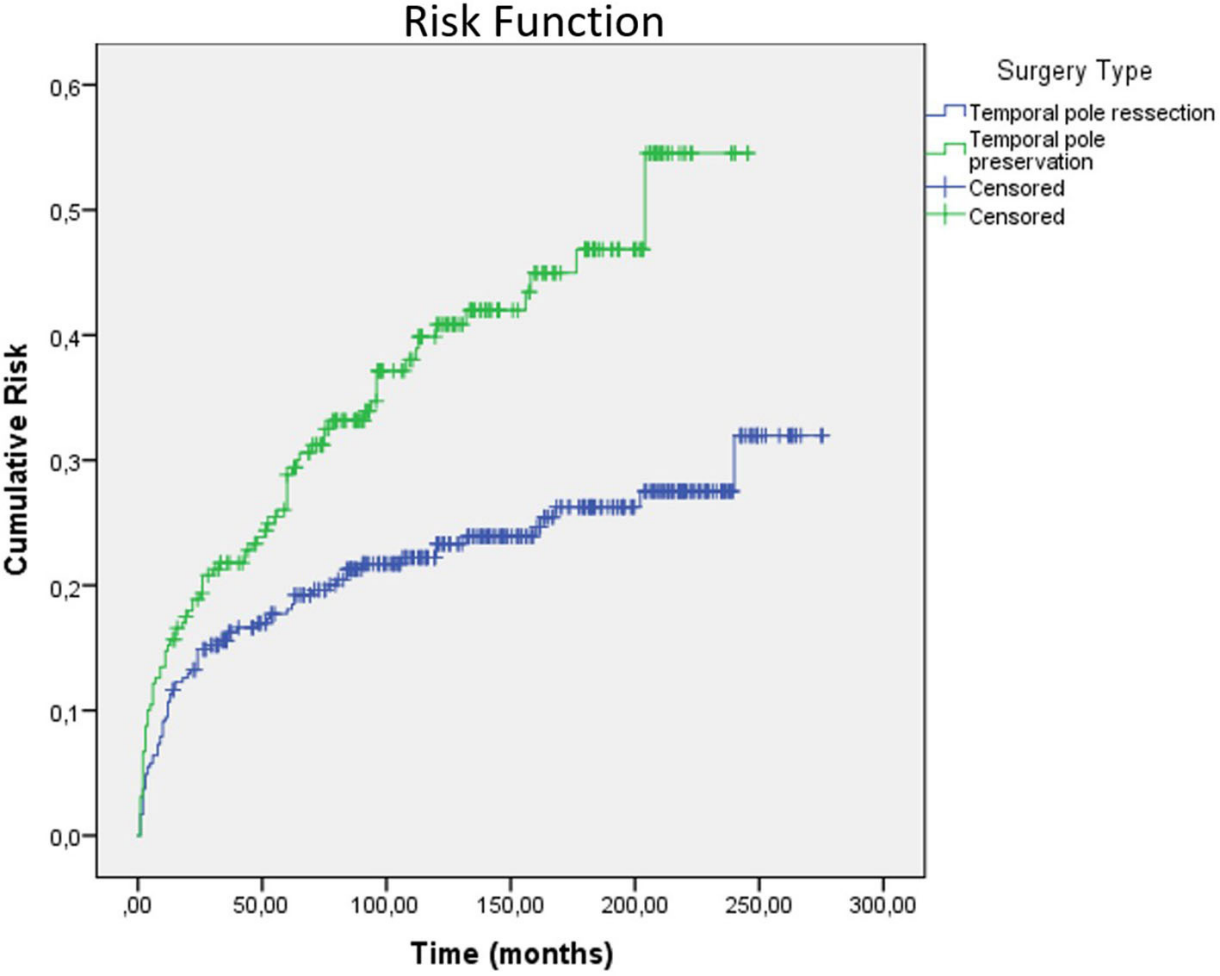
Risk function according to the type of surgery.

Factors associated with postsurgical seizure outcome were the type of surgical technique, history of a febrile seizure, presence of dysmnestic, or olfactory auras. Regarding the type of surgical technique, patients who underwent a standard ATL (with temporal pole resection) had a better outcome when compared to patients who underwent SAH *via* subtemporal approach, which preserves the temporal pole (78.6 *vs*. 67.2% Engel Class I, *p* = 0.002). When we analyze the Kaplan–Meier curve using the factor “type of surgery,” we observed that for patients who underwent ATL, the survival at 250–300 months follow-up was higher, when compared to patients submitted to SAH (Figures 4, 5).

Patients who reported a history of febrile seizures had a significantly better seizure outcome (79.4 vs. 71.4% of Engel I, *p* = 0.047). Patients who reported dysmnestic or olfactory auras had a worse seizure outcome (47.1 vs. 75% Engel Class I, *p* = 0.02/37.5 vs. 74.7% Engel Class I, *p* = 0.03, respectively).

### Binary Logistic Regression Analysis

We performed a multivariable regression model (binary logistic regression) to identify factors that predicted the outcome. The model revealed an ExpB = 3.627 (*N* = 621, *p* < 0.001), indicating that the model was able to distinguish between patients with a seizure-free. The predictor variables included in the model were type of surgery (ExpB = 0.553; *p* = 0.005), presence/absence of dysmnestic auras (ExpB = 0.328; *p* = 0.051), presence/absence of olfactory aura (ExpB = 0.137; *p* = 0.022), presence/absence of febrile seizure (ExpB = 1.605; *p* = 0.042). Through this model, we created the following equation:


$$\begin{array}{l} p=\frac{1}{\left\{1+e^{-\left[1.288-\left(0.592xSAH\right)-\left(1.114xDA\right)+\left(0.473xFC\right)-\left(1.986XOA\right)\right]}\right\}} \end{array}$$

where:

p = probability of being free from disabling seizures (Engel I);

e = natural log constant = 2.718;

SAH = selective amygdalohippocampectomy (preserves the temporal pole);

DA = dysmnestic aura;

FS = febrile seizure;

OA = olfactory aura;

The coefficients are: 0 = absence of factor, 1 = presence of factor.

### Non-significant Results

Variables that classically are known to influence the outcome, but did not influence the outcome in our study were (a) MRI with unilateral EMT versus bilateral EMT; (b) epigastric aura; (c) presence of bilateral or contralateral or extratemporal interictal discharges; (d) delay in neuropsychomotor development and low IQ; (e) history of frequent generalized tonic-clonic seizures; (f) age at surgery; (g) time of epilepsy and age at surgery. Other variables that also did not predict postsurgical seizure outcome included (a) gender; (b) presence of calcified cysticercosis lesions; (c) presence of an IPI (such as prolonged non-febrile seizure, status epilepticus, trauma brain injury, obstetric complications); (d) family history of febrile seizures; (e) family history of epilepsy; (f) presence of a focal neurological deficit; (g) lateralization signs in semiological, interictal, and ictal EEG; (h) having performed an invasive evaluation, and (i) side of surgery.

### Complications

Five hundred and forty patients had no immediate complications (87%; 95% *CI*: 84.1–89.5) and 537 patients had no late postoperative complications (86.5%; 95% *CI*: 83.5–89.1). The most common late complication was chronic headache 33 (5.3%; 95% *CI*: 3.7–7.4). Twenty-seven patients died during follow-up, of these 11 were not related to epilepsy (cancer, myocardial infarct, chronic renal disease). Fourteen patients of unknown causes (may or may not be sudden unexpected death in epilepsy) and two patients from traumatic brain injury associated with epileptic seizures. The immediate and late postoperative complications are summarized in Tables 4, 5.

**Table 4:** Immediate postoperative complications.

**Complications**	**N**	**Valid percentage**	**CI 95%**
None	540	87.0	84.1–89.5
Urinary tract infection	1	0.2	0–0.9
Erysipelas	2	0.3	0–1.2
Temporomandibular dysfunction	2	0.2	0–1.2
Psychiatric disorders	5	0.8	0.3–1.9
Epileptic seizure	17	2.7	1.6–4.3
Diplopia	7	1.1	0.5–2.3
Deep vein thrombosis	3	0.5	0.1–1.4
Otorrhagia	1	0.2	0–0.9
Ipsilateral hypoacusis	1	0.2	0–0.9
Skin flap necrosis	1	0.2	0–0.9
Meningitis	2	0.3	0.−1.2
Surgical wound infection	4	0.6	0.2–1.6
Intracranial bleeding	9	1.4	0.7–2.7
Stroke	5	0.8	0.3–1.9
Intraoperative cerebral edema	1	0.2	0–0.9
Nosocomial pneumonia	1	0.2	0–0.9
Language disorders	5	0.8	0.3–1.9
CSF fistula	3	0.5	0.1–1.4
No information	11	1.8	0.9–3.1
Total	621	100	

**Table 5:** Late postoperative complications.

**Late postoperative complications**	***N***	**Valid percentage**	**CI 95%**
None	537	86.5	83.5–89.1
Motor deficit	1	0.2	0–0.9
Chronic headache	33	5.3	3.7–7.4
Psychiatric Disorders	30	4.8	3.3–6.8
Stroke	5	0.8	0.3–1.9
Temporomandibular dysfunction	7	1.1	0.5–2.3
Brain abscess	1	0.2	0–0.9
Hydrocephalus	1	0.2	0–0.9
Cranial osteomyelitis	1	0.2	0–0.9
No information	5	0.8	0.3–1.9
Total	621		

### Reoperation

Of the 621 patients, 23 (3.7%; 95% *CI*: 2.4–5.5) were reoperated to expand the temporal lobectomy. Of the 23 patients who were reoperated, 18 (78%) had initially undergone SAH, and nine were seizure-free after the ATL procedure. Of these reoperated patients, 12 (52.2%) became free from disabling seizures (Engel Class I).

### Antiseizure Medication Withdraw

In the last consultation, 19.5% were able to completely withdraw ASMs and 35.9% were on monotherapy.

## Discussion

In this large series of 621 patients with TLE with a mean follow-up duration of 11.6 years, we found that 73.6% of the patients were Engel Class I. We observed that seizure outcome was sustained for the long term because 65% of patients were Engel Class I after more than 20 years of follow-up. Moreover, the rate of serious complications was low. This is the largest series with a longer follow-up duration. Our data confirm other series showing the sustained long-term benefit of temporal resection in medically refractory TLE (12, 13).

### Factors Associated With Seizure Outcome

#### Type of Surgery

We found a significantly better outcome regarding postsurgical seizure control in the group submitted to ATL when compared to SAH *via* subtemporal approach (Engel Class I - 78.6 vs. 67.2%, *p* = 0.002). The failure of SAH treatment suggests that the so-called “mesial-temporal seizures” may not always start in the amygdala–hippocampal complex. The epileptogenic zone may extend beyond the mesial temporal structures. The temporal pole is a paralimbic structure that is strongly connected with the amygdala, hippocampus, parahippocampal gyrus, cingulate gyrus, orbitofrontal cortex, and insula. Several groups have reported the importance of the temporal-polar cortex in the genesis of temporal lobe seizures (15, 16). In a study with 48 TLE patients evaluated by stereo EEG, in 48% of the cases, the temporal pole was involved in the seizure onset and 52% had late temporal pole involvement. Finally, in 35% of these patients with TLE-HS, seizures started at the temporal pole. The authors concluded that the involvement of the temporo-polar cortex in the onset of seizures may be the explanation for the failure of SAH in some cases (16). In addition, it is well known that in SAH, less neocortex is resected, which could also contribute to a poorer seizure outcome in patients submitted to more selected resections.

Finally, two meta-analyses found that patients who underwent ATL were more likely to remain seizure-free (Engel Class I) than patients who underwent SAH (17, 18). Other studies found no significant differences between these two groups (13, 19–21).

In the past, there were speculations that the presence of MRI findings of anterior temporal lobe atrophy with white matter abnormalities (usually called “blurring”) could represent dual pathology or cortical dysplasia, contributing to a poorer seizure outcome when not included in the temporal resection (22, 23). However, studies have shown that neuropathological reviews of the resected material showed that such anterior temporal pole blurring was not caused by dysplasia, degenerative abnormalities, or inflammatory changes, and may reflect a nonspecific increase in water content in the temporal lobe or myelin abnormalities (24). In addition, several studies have reported that the presence of temporal pole blurring did not influence the seizure outcome, even in patients submitted to temporal resections with temporal pole preservation (25, 26).

#### History of Febrile Seizures

Another finding of our study was that patients who reported a history of febrile seizures had a better seizure outcome (79.4 vs. 71.4% of Engel I, *p* = 0.047), which has been described (27, 28). However, many studies fail to control for clinical factors that are highly correlated. For example, a meta-analysis studying predictors of seizure outcome in patients with TLE concluded that a history of febrile seizures was associated with good post-surgical outcomes (27). It is important to consider that most studies that reported this association pooled patients with TLE-HS and patients with normal MR (12, 29). This could explain such association because in patients with TLE-HS, who have a better outcome than those with normal MRI, a history of febrile convulsion is more common than in patients with normal MRI. Also, most studies analyzing exclusively patients with TLE-HS reported no association between febrile seizures and good postsurgical outcomes (29, 30). What could explain such association in our study, reporting a better outcome in patients with a history of febrile seizures, was our much larger sample size.

#### Auras

In our study, we observed a significantly worse outcome in patients who reported an olfactory aura. This finding could be explained by the complex connections involved by the olfactory tract and by the location of the primary olfactory cortex, such as the orbitofrontal cortex, located in the basal frontal lobe and piriform cortex. Thus, it is suggested that the olfactory aura may not only be related to the symptomatic zone but may also be involved with the ictal onset zone in areas adjacent to the temporal lobe (31). We also observed a significantly worse outcome in patients who reported dysmnestic auras. We did not find data in the literature that corroborate our findings.

#### The Binary Logistic Regression Model

The binary logistic regression model allowed the estimation of the likelihood of becoming seizure-free after surgery. For example, using the formula described in the Results section, a patient with a history of febrile seizures, no dysmnestic aura, no olfactory aura, submitted to ATL would have the probability of becoming seizure-free of 85%, compared to 8.3% in a patient without a history of febrile seizures, a history of dysmnestic aura and olfactory aura, submitted to SAH. Binary logistic regression could help the presurgical evaluation team in decision-making, and the opportunity to clarify to the patient more accurately their probability of staying seizure-free after surgery.

#### Why Bilateral HS Was Not a Predictor of Seizure Outcome?

It is classically described that patients with unilateral HS had a better surgical outcome when compared to patients with bilateral HS regardless of the type of surgical technique adopted (19). In our study, we did not observe any significant difference regarding the surgical outcome between the groups of patients with unilateral vs. bilateral HS, regardless of the type of surgery. The number of patients in the group with unilateral HS was significantly higher, 96.6% (*n* = 600), while the number of patients with bilateral HS was much lower, 3.3% (*n* = 21). Also, patients with bilateral HS who during phase I evaluation had bilateral or contralateral ictal EEG were submitted to an invasive (SEEG or subdural electrodes) or semi-invasive (foramen ovale electrodes) evaluation. In these cases, if the invasive evaluation showed unilateral seizures, the patients were submitted to surgery. In cases where the invasive evaluation was bilateral, surgery was contraindicated, which explains why the sample size of patients with bilateral HS who underwent surgery was low, as we probably contraindicated many surgeries with bilateral EEG onsets on invasive evaluation. Therefore, our surgical outcome was similar between these two groups.

### Reoperation

In our study, we found evidence that in patients with seizure recurrence after SAH, 50% became seizure-free after subsequent ATL. Our findings are in line with previous studies showing a significant improvement in the post-surgical seizure outcome after subsequent ATL in patients in which the initial SAH failed to control the seizures, where 69.2% of patients were seizure-free (Engel Class I). The study also concluded that the risk of neuropsychological decline after the second surgery was low and that the improvement in seizure control after the second surgery had a positive influence on cognitive performance (32). This can be a valuable criterion for advising eligible patients for a second surgery regarding the risks of cognitive deficits.

## Conclusion

Our large database confirms the sustained long-term benefit of temporal resection in medically refractory TLE-HS with low rates of postoperative complications. Two-thirds of patients with a follow-up duration of more than 20 years were seizure-free. We also found that ATL significantly improved the chances of being seizure-free in the long term when compared to more limited resection (SAH). Finally, if suspected of drug-resistant temporal lobe epilepsy, it is recommended to refer the patient to an epilepsy surgery center for pre-surgical evaluation.

## Data Availability Statement

The raw data supporting the conclusions of this article will be made available by the authors, without undue reservation.

## Ethics Statement

The studies involving human participants were reviewed and approved by Research Ethics Committee of Hospital of Clinics of the Medical School of Ribeirão Preto of the University of São Paulo (HCFMRP-USP). Written informed consent for participation was not required for this study in accordance with the national legislation and the institutional requirements.

## Author Contributions

The authors have contributed significantly to pre-surgical evaluation team, discussion of clinical cases, surgical decision, patient follow-up, analysis, interpretation of data, and approved it for publication.

## Conflict of Interest

The authors declare that the research was conducted in the absence of any commercial or financial relationships that could be construed as a potential conflict of interest.

## Publisher's Note

All claims expressed in this article are solely those of the authors and do not necessarily represent those of their affiliated organizations, or those of the publisher, the editors and the reviewers. Any product that may be evaluated in this article, or claim that may be made by its manufacturer, is not guaranteed or endorsed by the publisher.
